# Norcycloartocarpin targets Akt and suppresses Akt-dependent survival and epithelial-mesenchymal transition in lung cancer cells

**DOI:** 10.1371/journal.pone.0254929

**Published:** 2021-08-12

**Authors:** Nongyao Nonpanya, Kittipong Sanookpan, Keerati Joyjamras, Duangdao Wichadakul, Boonchoo Sritularak, Chatchai Chaotham, Pithi Chanvorachote

**Affiliations:** 1 Department of Pharmacology and Physiology, Faculty of Pharmaceutical Sciences, Chulalongkorn University, Bangkok, Thailand; 2 Faculty of Pharmaceutical Sciences, Chulalongkorn University, Bangkok, Thailand; 3 Graduate Program of Pharmaceutical Sciences and Technology, Faculty of Pharmaceutical Sciences, Chulalongkorn University, Bangkok, Thailand; 4 Department of Computer Engineering, Faculty of Engineering, Chulalongkorn University, Bangkok, Thailand; 5 Departments of Pharmacognosy and Pharmaceutical Botany, Chulalongkorn University, Bangkok, Thailand; 6 Department of Biochemistry and Microbiology, Faculty of Pharmaceutical Sciences, Chulalongkorn University, Bangkok, Thailand; Chung Shan Medical University, TAIWAN

## Abstract

In searching for novel targeted therapeutic agents for lung cancer treatment, norcycloartocarpin from *Artocarpus gomezianus* was reported in this study to promisingly interacted with Akt and exerted the apoptosis induction and epithelial-to-mesenchymal transition suppression. Selective cytotoxic profile of norcycloartocarpin was evidenced with approximately 2-fold higher IC_50_ in normal dermal papilla cells (DPCs) compared with human lung cancer A549, H460, H23, and H292 cells. We found that norcycloartocarpin suppressed anchorage-independent growth, cell migration, invasion, filopodia formation, and decreased EMT in a dose-dependent manner at 24 h, which were correlated with reduced protein levels of N-cadherin, Vimentin, Slug, p-FAK, p-Akt, as well as Cdc42. In addition, norcycloartocarpin activated apoptosis caspase cascade associating with restoration of p53, down-regulated Bcl-2 and augmented Bax in A549 and H460 cells. Interestingly, norcycloartocarpin showed potential inhibitory role on protein kinase B (Akt) the up-stream dominant molecule controlling EMT and apoptosis. Computational molecular docking analysis further confirmed that norcycloartocarpin has the best binding affinity of -12.52 kcal/mol with Akt protein at its critical active site. As Akt has recently recognized as an attractive molecular target for therapeutic approaches, these findings support its use as a plant-derived anticancer agent in cancer therapy.

## Introduction

Despite several decades of research and development for novel therapies, lung cancer still possesses the highest mortality rate among various types of cancer all over the world [1, 2]. The aggressive features including chemotherapeutic resistance contribute to poor prognosis and recurrence of tumor pathology consequence with low 5-year survival rate in lung cancer patients [3, 4]. The low susceptibility to available chemotherapies has been continuously documented in lung cancer cells especially the isolation from patients [5–9]. These therapeutic failures of current anticancer drugs provoke the seeking for an effective chemotherapy for lung cancer treatment [10, 11].

Recent studies have shown that epithelial-mesenchymal transition (EMT) plays an important role in cancer progression, metastasis, and resistance to chemotherapy [12–14]. During metastasis, EMT is an important cellular process through which epithelial cells acquire the migratory and invasive properties of mesenchymal cells [15]. EMT has been implicated in cancer metastasis by increasing anoikis resistance, as well as enhancing cell survival in anchorage-independent growth conditions [16, 17]. The critical hallmarks of EMT are epithelial cell markers (E-cadherin, ZO-1, and cytokeratin), mesenchymal cell markers (N-cadherin and vimentin), and EMT-related transcription factors such as twist, snail, slug, ZEB1 and ZEB2 proteins [18–20]. Moreover, activation of the Akt is an important signaling pathways that regulates critical cellular processes including cell proliferation, migration, survival, cancer progression and metastasis through phosphorylation of downstream targets [21, 22].

Evading from apoptosis induced by chemotherapeutic agents has been recognized as one of major drug resistant mechanisms [23, 24]. Indeed, apoptosis is a program cell death that involves in organ development and tissue homeostasis [25]. Moreover, most of novel anticancer agents potentially stimulate apoptosis cascade in order to eradicate highly proliferative tumor cells [26–28]. The alteration of proteins in Bcl-2 family effectively modulates apoptosis in lung cancer cells [29]. Clinical specimens from lung cancer patients demonstrate the up-regulation of B-cell lymphoma 2 (Bcl-2), an anti-apoptosis protein that plays a crucial role in drug resistance [30–32]. Currently, targeting on survival pathway mediated by protein kinase B (Akt) has been highlighted for lung cancer treatment [33, 34]. Down-regulated Akt-survival signal associating with the activation of tumor suppressor p53 protein shows a promising therapeutic effect in various cancers [35, 36]. Restoration of p53 function also intensifies the expression of Bcl-2-associated X (Bax), a pro-apoptosis protein which triggers executive caspase-3 and eventually apoptosis induction [37, 38].

**Fig 1:**
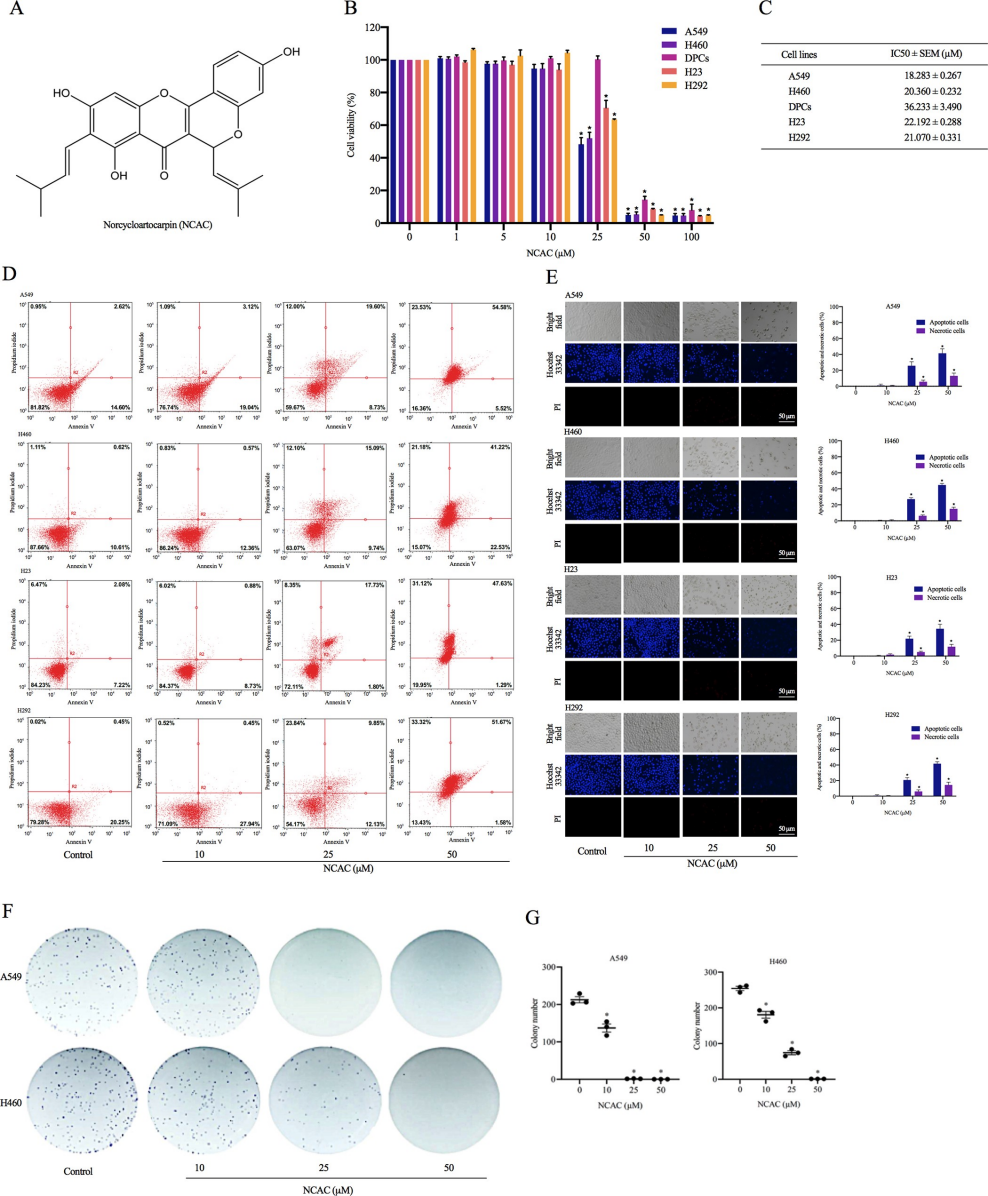
The selective anticancer activity of (**A**) norcycloartocarpin (NCAC), a flavonoid extracted from *A*. *gomezianus* was indicated by (**B**) the significant reduction of %cell viability in human lung cancer A549, H460, H23, and H292 cells while no alteration found in human dermal papilla DPCs cells cultured with NCAC at 10–25 μM for 24 h. (**C**) The half maximal inhibitory concentration (IC_50_) of NCAC in DPCs cells was approximately 2-fold higher than the IC_50_ in lung cancer A549, H460, H23, and H292 cells. (**D**) Annexin V/PI co-stained cells were examined using flow cytometry. (**E**) Apoptotic and necrotic A549 and H460 cells were detected by Hoechst 33342/PI staining and visualized by fluorescence microscopy. Percentage of apoptotic/necrotic nuclei in NCAC-treated cells was analyzed. (**F**) the inhibitory effect of NCAC on colony formation in human lung cancer cells. The ability to form a new cancer colony was evaluated through clonogenic assay in viable single cell of lung cancer cells after culture with NCAC at 10–50 μM for 24 h. (**G**) The amount of forming colony was significantly decreased in NCAC-treated A549 and H460 cells compared with non-treated control cell. Data are represented as mean ± SEM from three independent experiments. *p < 0.05 versus non-treated control cells.

Not only drug resistance but also serious side-effects restrict the usage of current chemotherapies [39]. Emerging evidence demonstrate high anticancer potency of diverse natural compounds with human safety profile [40]. Intriguingly, the flavonoids extracted from various plants exhibit the suppressive effect against human lung cancer cells [41–43]. Nevertheless, anticancer activity of norcycloartocarpin (Fig 1A), a flavonoid derivative from *A*. *gomezianus* Wall. ex Tréc. (Moraceae), a Thai medicinal plant, has never been reported. Herein, apoptotic induction and anti-migration effects of norcycloartocarpin were investigated in human lung cancer cells. The obtained information would facilitate the development of norcycloartocarpin as a novel chemotherapeutic agent for lung cancer.

## Materials and methods

### Preparation of norcycloartocarpin

Norcycloartocarpin was isolated from the heartwood of *A*. *gomezianus* as previously described [44]. Norcycloartocarpin with more than 95% purity determined by NMR spectroscopy was used in this study. The chemical structure of norcycloartocarpin shown as Fig 1A, it was dissolved in dimethyl sulfoxide (DMSO) (Sigma Chemical, St. Louis, MO, USA) and then stored at -20°C. The stock solution was diluted with culture medium to achieve the desired concentrations. The final concentration of DMSO was less than 0.5%, which showed no toxicity towards the tested cells.

### Cell culture and chemical reagents

Human lung cancer A549, H460, H23, and H292 cells were obtained from the American Type Culture Collection (Manassas, VA, USA) and cultured in Dulbecco’s Modified Eagle’s Medium (DMEM) and Roswell Park Memorial Institute (RPMI) 1640 medium (Gibco, Grand Island, NY, USA), respectively. Meanwhile, human dermal papilla DPCs cells from Applied Biological Materials Inc. (Richmond, BC, Canada) were maintained in Prigrow III medium (Applied Biological Materials Inc., Richmond, BC, Canada). All culture mediums were supplemented with 10% FBS, 100 units/ml penicillin/streptomycin and 2 mM L-glutamine. The cells were maintained in humidified incubator containing 5% CO_2_ at 37°C until reached 70% confluence before using in further experiments. Hoechst33342, propidium iodide (PI), dimethyl sulfoxide (DMSO), phosphate-buffered saline (PBS; pH 7.4), MTT (3-(4,5-dimethylthiazol-2-yl)-2,5-diphenyltetrazolium bromide), Phalloidin-Rhodamine, and bovine serum albumin (BSA) were purchased from Sigma Chemical, Inc. (St. Louis, MO, USA). Annexin V-FITC apoptosis kit was purchased from Thermo Fisher Sciencetific (Waltham, MA, USA). Antibody for poly ADP ribose polymerase (PARP), caspase-9, caspase-3, Bcl-2, Bax, p53, phosphorylated Akt (p-Akt; Ser 473), Akt, p-FAK (p-FAK; Tyr 397), FAK, Slug, Snail, Vimentin, N-cadherin, E-cadherin, β-actin, as well as the respective secondary antibodies were purchased from Cell Signaling Technology (Danvers, MA, USA).

### Cell viability assay

Cell viability was determined by MTT assay, which detects the cellular capacity to reduce MTT to formazan crystals by mitochondria dehydrogenase. Cells were seeded onto 96-well plates (1 × 10^4^ cells/well) for overnight. After the further culture with various concentrations of norcycloartocarpin (0–100 μM) for 24 h, the culture medium was replaced with 100 μl/well of MTT solution (0.4 mg/ml) and then incubated at 37°C for 3 h in dark place. Subsequently, MTT solution was removed before adding 100 μl/well of DMSO to dissolve the formazan crystal. The optical density was measured at 570 nm using a microplate reader (Anthros, Durham, NC, USA). All analyses were performed in at least three independent replicate culture. The ratio of optical density between treated to non-treated cells was calculated and presented as percentage of cell viability.

### Detection of mode of cell death

After treatment with different concentrations of norcycloartocarpin for 24 h, lung cancer A549, H460, H23, and H292 cells at a density of 1 × 10^4^ cells/well in 96-well plates were evaluated for mode of cell death. Apoptosis and necrosis cells were determined by nuclear co-staining with 10 μM of Hoechst 33342 and 5 μM of propidium iodide (PI) for 15 min at 37°C protected from light. The cells were visualized and imaged by using a fluorescence microscopy (ECLIPSE Ts2; Nikon). Three random fields were captured at 20× magnification and then the percentages of apoptosis and necrosis cells were calculated.

### Flow cytometry analysis

A549, H460, H23, and H292 cells were seeded onto 96-well plates and incubated at 37°C for overnight. Cells were treated with norcycloartocarpin at concentrations 10, 25, and 50 μM, and incubated at 37°C for 24 h. Apoptotic and necrotic were determined using Annexin V-FITC/PI apoptosis kit (Thermo Fisher Sciencetific, Waltham, MA, USA). Cells were suspended with 100 μl of 1× binding buffer and incubated with 5 μl of PI in the dark at room temperature for 15 min. Subsequently, binging buffer added 400 μl of incubation buffer, and cells were analyzed using guava easyCyteTM flow cytometry (Merk, DA, Germany).

### Colony forming assay

Survival ability and capability to form a new colony of single cancer cell were examined through clonogenic or colony forming assay [45]. The single cell suspension containing 250 viable cells was prepared from human lung cancer cells after treatment with 0–50 μM norcycloartocarpin for 24 h. The cells were placed onto 6-well plate and further cultured under 5% CO_2_ at 37°C for 7 days. Then, forming colonies were stained with 0.05% w/v crystal violet in 4% formaldehyde for counting the colony number.

### Anchorage-independent growth assay

A549 and H460 cells were pre-treated with non-cytotoxic concentration of norcycloartocarpin (0–10 μM) for 24 h and assigned to anchorage-independent growth assay. Soft agar colony-formation assay was used to investigate anchorage-independent cell growth. The bottom layer was prepared by using a 1:1 mixture of DMEM or RPMI1640 medium containing 1% FBS (Merck, DA, Germany) and 1% agarose, then was allowed to solidify for 20 min at 4°C. The upper layer consisting of 0.3% agarose gel with 1% FBS (Merck, DA, Germany) and containing 1 × 10^3^ cells/ml was prepared and added. DMEM or RPMI1640 medium containing 1% FBS (Merck, DA, Germany) was added over upper layer. Cells were incubated for 14 days at 37°C and colony formation was counted and captured using a phase-contrast microscope (Nikon ECLIPSE Ts2, Tokyo, Japan). Relative colony number and size were counted and calculated by dividing the values of the treated cells by the non-treated cells.

### Migration and invasion assay

Cell migration was determined using wound healing assay. 2 × 10^4^ cells were cultured in each well of 96-well plates, and were created wound space by micropipette tip. After that, media was removed and washed with phosphate buffered saline (PBS) (Gibco, Grand Island, NY, USA). The cell monolayers were incubated with non-toxic concentration of norcycloartocarpin (0–10 μM), and permitted to migrate for 24 and 48 h. Under a phase contrast microscope, the photos of cell migration were taken and were measured wound space using Image J software (NIH, Bethesda, MD, USA). The percentage of the changed wound space was calculated as follows: Change in the wound space (%) = (average space at time (0–24 h, 48 h)/ average space at time 0 h) × 100. Relative cell migration was calculated by dividing the percentage change in the wound space of treated cells by that of the control cells in each experiment.

Additionally, cell invasion assay was performed using a transwell Boyden chamber (8 μm pore size; BD Bioscience, MA, USA). The upper chamber was coated with 0.5% Matrigel (BD Biosciences, San Jose, CA, USA) overnight at 37°C. A549, H460, H23, and H292 cells were pre-treated with norcycloartocarpin at non-toxic concentrations (0–10 μM) for 24 h at 37°C. The treated cells were seeded at a density of 2 × 10^4^ cells/well in the upper chamber supplemented with serum free medium, while the complete medium containing 10% FBS (Merck, DA, Germany) was added to the lower chamber compartment as a chemoattractant. After incubation, the non-invading cells in the upper chamber were removed with a cotton swab and the invading cells in the lower chamber were fixed with cold methanol (Merck, DA, Germany) for 10 minutes and stained with 10 μM of Hoechst 33342 (Sigma, St. Louis, MO, USA) for 10 minutes. Finally, the stained cells were visualized and captured using a fluorescence microscope (Nikon ECLIPSE Ts2, Tokyo, Japan). Relative migration or invasion were calculated by dividing the number of migrated cell or invaded cells treated with norcycloartocarpin compared to the non-treated cells in each experiment.

### Cell morphology and filopodia characterization

Cell morphology and filopodia were investigated by a phalloidin-rhodamine staining assay. A549 and H460 cells were seeded in 96-well plates for overnight and then treated non-toxic concentration of norcycloartocarpin for 24 h. After treatment, norcycloartocarpin-treated A549 and H460 cells were fixed with 4% paraformaldehyde (Sigma Chemical, St. Louis, MO, USA) for 10 min at 37°C. Then, cells were permeabilized with 0.1% Triton X (Sigma Chemical, St. Louis, MO, USA) in PBS (Gibco, Grand Island, NY, USA) for 5 min and blocked for unspecific binding with 0.2% BSA (Merck, DA, Germany) for 30 min, incubated with a 1:100 dilution of phalloidin-rhodamine (Sigma Chemical, St. Louis, MO, USA) in PBS (Gibco, Grand Island, NY, USA) for 30 min, rinsed in PBS (Gibco, Grand Island, NY, USA) three times and mounted in 50% glycerol in PBS (Gibco, Grand Island, NY, USA). Cell morphology changes and filopodia were visualized and captured using a fluorescence microscopy (Nikon ECLIPSE Ts2, Tokyo, Japan). Relative number of filopodia/cell was determined the number of filopodia/cell of the norcycloartocarpin-treated cells dividing by control cells.

### Western blot analysis

After treatment with norcycloartocarpin for 24 h, A549, H460, H23, and H292 cells were harvested and washed twice with cold PBS. The cells were lysed with radioimmunoprecipitation assay (RIPA) lysis buffer containing a protease inhibitor cocktail (Roche Diagnostics, Indianapolis, IN, USA) for 1 h on ice. The protein concentration of cell lysate was determined using a bicinchoninic acid (BCA) protein assay kit (Pierce, Rockford, IL, USA). All protein samples were run on sodium dodecyl sulfate polyacrylamide gel electrophoresis (SDS-PAGE) and further transferred onto 0.45 μm nitrocellulose membranes (Bio-Rad, Laboratories, Hercules, CA, USA). Then, immersion of the membranes into 5% non-fat dry milk in TBST (25 mM Tris-HCl pH 7.5, 125 mM NaCl, and 0.05% Tween-20) was performed at room temperature for 1 h to prevent non-specific binding. The membranes were further incubated with the specific primary antibodies against PARP, caspase-9, caspase-3, Bcl-2, Bax, p53, p-Akt, Akt, Slug, Snail, Vimentin, N-cadherin, E-cadherin, p-FAK, FAK, and β-actin at 4°C for overnight. Subsequently, the membranes were washed three times with Tris-buffered saline containing with Tween 20 and then incubated with horseradish peroxidase (HRP)-conjugated secondary antibody for 2 h at room temperature. Immunoreactive proteins were detected with the enhanced chemiluminescent detection system (Supersignal West Pico, Pierce, Rockford, IL, USA) and imaged with ImageQuant LAS 4000 biomolecular imager (GE Healthcare, Chicago, Illinois, United States). The intensity of specific protein band was analyzed using the ImageJ software (version 1.52, National Institutes of Health, Bethesda, MD, USA).

### RNA isolation, reverse transcription and quantitative real-time PCR (qRT-PCR)

A549 and H460 cells were treated with non-toxic concentration of norcycloartocarpin for 24h, and total RNA was extracted from the treated cells using the RNeasy Mini kit according to the protocol of the manufacturer (Qiagen GmbH, Hilden, Germany). RNA was reverse transcribed using a kit from Qiagen (QIAGEN Inc., Maryland, USA), then cDNA was diluted with distilled RNAse/DNase free water. 5 μl of cDNA (20 ng) was used per reaction. The thermocycling conditions were as follows: 95°C for 15 min; and 50 cycles at 94°C for 20 s, 50°C for 30 sec, and 72°C for 20 sec. The primers were as follows: N-cadherin forward primer, 5’-GACCGAGAATCACCAAATGTG-3’, reverse primer, 5’-GCGTTCCTGTTCCA CTCATAG-3’; Vimentin forward primer, 5’-ACCCTGCAATCTTTCAGACAG-3’, reverse primer, 5’-GATTCCACTTTGCGTTCAAGG-3’; Slug forward primer, 5’-AGCATTTCA ACGCCTCCA-3’, reverse primer, 5’-GGATCTCTGGTTGTGGTATGAC-3’; β-actin forward primer, 5’-AGAGCTACGAGCTGCCTGAC-3’ and reverse primer, 5’-AGCACTGTGTTGGCGTACAG-3’. The expression level of each gene of interest was normalized to the β-actin. The data were calculated using the ΔΔC_t_ method. Each sample was performed in triplicate.

### Immunofluorescence assay

A549 and H460 cells were seeded onto 96-well plates at a density 1 × 10^4^ cells/well and incubated overnight. The cells were treated with non-toxic concentration of norcycloartocarpin for 24 h. The cells were washed twice with 1 × PBS and fixed with 4% paraformaldehyde for 20 min, permeabilized with 0.1% Triton-x in PBS for 20 min, and blocked with 4% BSA in 1 × PBS for 30 min at room temperature. The cells were incubated with primary antibodies (N-cadherin, vimentin, and p-Akt) at 4°C overnight, the cells were washed twice with 1 × PBS and incubated with secondary antibodies for 1 h in the dark at room temperature. The treated cells were washed with 1 × PBS and incubated with Hoechst 33342 (Sigma, St. Louis, MO, USA) for 20 min in the dark, rinsed with 1 × PBS and mounted by 50% glycerol (Merck, DA, Germany). Confocal images were assessed under fluorescence microscope with a 40× objective lens (Nikon ECLIPSE Ts2, Tokyo, Japan) and the analysis was assessed by ImageJ software.

### Computational Akt modelling and molecular docking

The binding affinity of norcycloartocarpin to important Akt protein and regulators crucial for EMT and apoptosis in human cancer cells was determined using molecular docking methods. To prepare for the docking study, the crystal structures of human Akt (PDB ID: 4EKL) [46] was obtained from Protein Data Bank (PDB). The structure of norcycloartocarpin was downloaded from PubChem and converted into pdb format using openbabel. Both pdb files were then converted to pdbqt format using AutoDockTools [47]. We then used AutoDock vina to perform the docking calculation of the norcycloartocarpin to the ATP binding pocket [48]. For the molecular dynamics (MD) simulations, the missing amino acid residues of 4EKL were completed using the Swiss-PdbViewer [49]. We used Avogadro [50] to add hydrogens to norcycloartocarpin and used ACPYPE-AnteChamber [51] to generate the mol2 and topology files. We applied the general AMBER force field (GAFF) [52] for the ligand and the AMBER ff14SB force field for the protein [53]. The system was then solvated using TIP3P water model [54]. The Na^+^ and Cl^-^ ions were added to neutralize the system. The steepest descent was used for energy minimization. We used V-rescale [55] for temperature coupling with coupling constant of 0.1 ps. Th electrostatic and van der Waals interactions were based on the Particle Mesh Ewald (PME) algorithm [56]. The short-range van der Waals (rvdw) electrostatic (rcoulomb) cutoffs and neighbor list (rlist) were set to 12 angstroms. The LINCS algorithm was used to constrain all bond lengths [57]. The time step was set to 0.002 ps. The complex was equilibrated in NVT and then NPT ensembles, each with 100 ps. The molecular dynamics (MD) simulation based on GROMACS 2020.4 was then carried out for 300 ns [58, 59]. The heavy-atom of norcycloartocarpin was then measured for the root mean square deviation (RMSD). The binding free energies were calculated based on MM/GBSA method [60] via the gmx_MMPBSA program [61]. We then evaluated the per-residue decomposition to identify the key amino acids for ligand recognition. The PyMOL molecular graphics program (Schrödinger, Inc.) was utilized for general structural representation of the FERM domain and publication-quality images were made using the ray-trace command.

### Statistical analysis

The data from three independent experiments (n = 3) are presented as the mean ± standard error of the mean (SEM). Statistical differences between multiple groups were analyzed using an analysis of variance (ANOVA) and *post-hoc* test at a significance level of *p* < 0.05. Graphpad Prism version 9 (Graphpad Software, San Diego, CA, USA) was used to analyze all data.

## Results

### Selective cytotoxicity of norcycloartocarpin in human lung cancer cells

The incubation of human lung cancer A549, H460, H23, and H292 cells with norcycloartocarpin (0–100 μM) extracted from *A*. *gomezianus* for 24 h resulted in the reduction of viable cells in dose-dependent manner (Fig 1B). The significant diminution of % cell viability in A549, H460, H23, and H292 cells was presented at the low concentration (10 μM) of norcycloartocarpin compared with the non-treated control cells. The comparable cytotoxic activity against various human lung cancer cells of norcycloartocarpin was indicated with the half maximal inhibitory concentration (IC_50_) at 18.283 ± 0.267, 20.360 ± 0.232, 22.192 ± 0.288, and 21.070 ± 0.331 μM in A549, H460, H23, and H292 cells, respectively (Fig 1C). Interestingly, the selective cytotoxicity of norcycloartocarpin was evidenced with the higher IC_50_ (36.233 ± 3.490 μM) in human dermal papilla DPCs cells which is one of the most affected normal cells in chemotherapeutic treatment [62, 63]. Although norcycloartocarpin at 50 μM dramatically decreased viability in all selected cells, the further investigations were performed in human lung cancer cells treated with 0–50 μM norcycloartocarpin to clearly clarify the regulatory mechanisms involving in anticancer activity.

In order to investigate cytotoxic activity of norcycloartocarpin in human lung cancer cells, nuclear staining assay was performed for detection of mode of cell death. The alteration of cell morphology was remarkably observed in human lung cancer cells after culture with norcycloartocarpin at 10, 25, and 50 μM for 24 h. Additionally, staining with Hoechst33342 clearly demonstrated apoptosis cells presenting bright blue fluorescence of condensed DNA and/or fragmented nuclei in norcycloartocarpin-treated A549, H460, H23, and H292 cells (Fig 1E). We found that a norcycloartocarpin at 10 μM had no effect on apoptosis or necrosis, whereas norcycloartocarpin at 25 and 50 μM induced apoptosis in A549, H460, H23, and H292 cells (Fig 1E). The augmented apoptosis was concentration dependent and reached approximately to 50% in A549, H460, H23, and H292 cells cultured with 25 μM norcycloartocarpin. It was worth to note that the late stage of apoptosis that exhibit both bright blue of Hoechst 33342 and red fluorescence of PI-stained nuclei were detected in A549, H460, H23, and H292 cells cultured with 25 and 50 μM norcycloartocarpin. The results shown that norcycloartocarpin induced apoptosis at a concentration of 25 and 50 μM, while necrosis was rarely detected in all the treated conditions. To confirm, apoptosis and necrosis in response to norcycloartocarpin treatment were detected by annexin V/PI assay and analyzed using flowcytometry. The result showed that norcycloartocarpin at 10 μM did not cause either apoptosis or necrosis in these cells at 24 h (Fig 1D). We also found that norcycloartocarpin at concemtrations of 25 and 50 μM induced apoptosis. Non-toxic (10 μM) and toxic (25 and 50 μM) concentrations of norcycloartocarpin were used for the further experiments.

### Norcycloartocarpin restrains colony formation in human lung cancer cells

The effect of down-regulated Akt on survival of norcycloartocarpin-treated lung cancer cells was further evaluated. Viable A549 and H460 cells derived from the culture with norcycloartocarpin (10, 25, and 50 μM) for 24 h were subjected to clonogenic assay. The crystal violet-staining colony which represents the capability to survive and reproduce a new cancer colony from single cell of human lung cancer A549 and H460 cells was obviously demonstrated in Fig 1F and 1G, respectively. Surprisingly, the suppression on colony regeneration was significantly indicated in both A549 and H460 cells obtained after the incubation with 10–50 μM norcycloartocarpin. The reduction of colony number was concentration dependent in both human lung A549 and H460 cells (Fig 1G). The inhibition on colony formation was correspondent with the diminution of p-Akt/Akt expression level in norcycloartocarpin-treated lung cancer cells. It should be noted the forming colony was hardly detected in the cells treated with 50 μM of norcycloartocarpin.

### Norcycloartocarpin suppressed cell migration and invasion, and anchorage-independent growth

**Fig 2:**
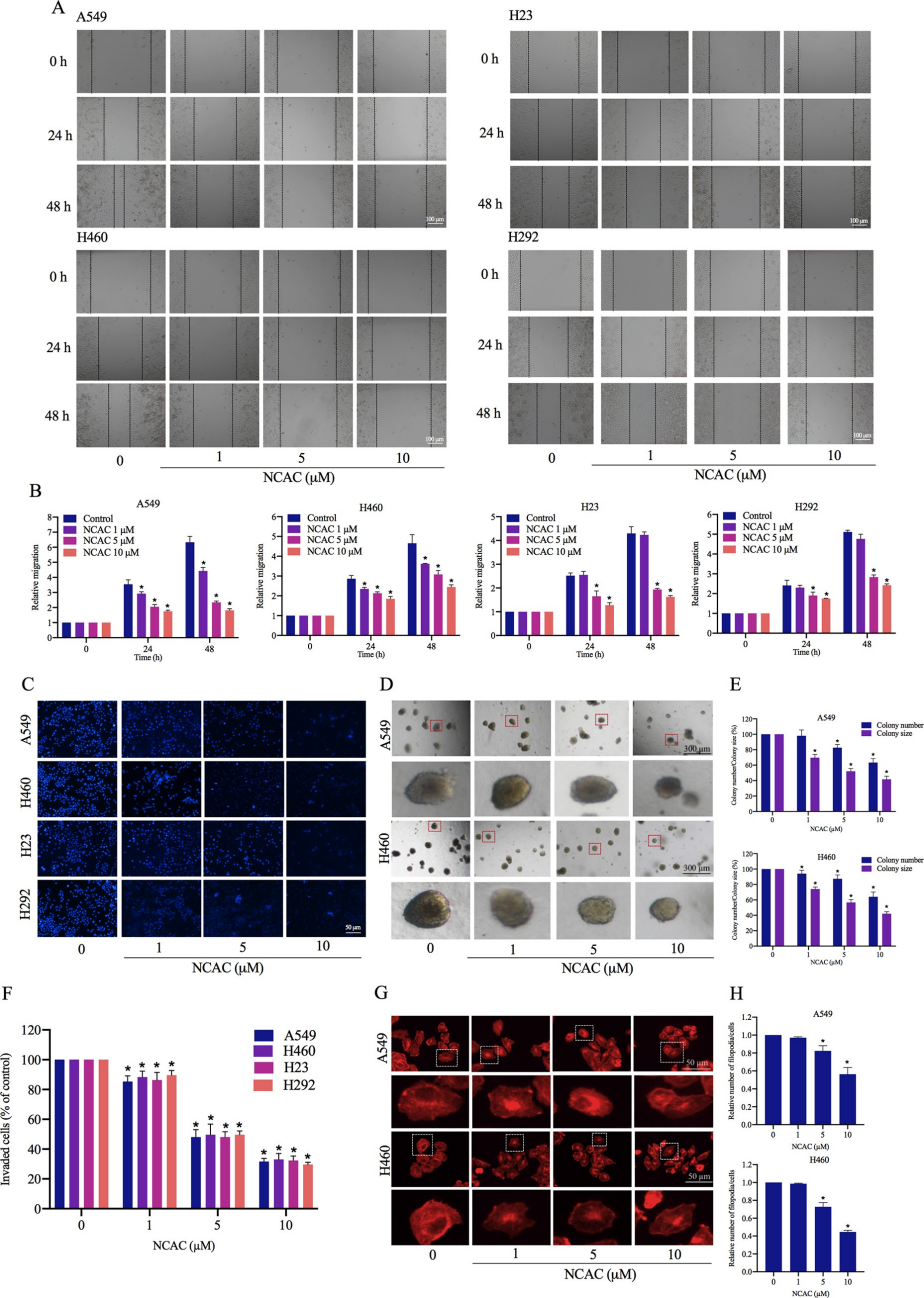
Norcycloartocarpin (NCAC) supresses anchorage-independent growth, cell invasion, migration, and filopodia. (**A**, **B**) NCAC decreased A549, H460, H23, and H292 cell migration: Cells were treated with non-toxic doses of NCAC (1, 5, and 10 μM), and migrations at 24 and 48 h were investigated. The relative cell migration was determined by comparing with the control. (**C**, **F**) cell invasion was determined using transwell invasion assay. The relative cell migration and invasion were investigated by comparing to control. After 24 h, the invaded cells were stained with Hoechst 33342 and visualized by fluorescence microscopy. The relative invasion level was calculated as the number of invaded cells of the treatment groups divided by that of the untreated control group. (**D**, **E**) Cells were pre-treated with non-toxic doses of NCAC (1, 5, and 10 μM) for 24 h, and were subjected to an anchorage-independent growth assay. (**G**, **H**) Effect of NCAC on filopodia formation: After treatment with non-toxic concentrations of NCAC for 24 h, cells were stained with phalloidin-rhodamine and examined using fluorescent microscopy. Relative number of filopodia per cell in A549 and H460 cells treated with NCAC, compared with the control. Data represent the mean ± SD (n = 3). *p<0.05 versus non-treated control.

Cell migration was determined by a wound-healing assay, whereby monolayers of A549, H460, H23, and H292 cells were treated with norcycloartocarpin (1, 5, and 10 μM) for 24 and 48 h. The results showed that norcycloartocarpin significantly inhibited A549 and H460 cell migration at the concentrations of 1, 5, and 10 μM at 24 and 48 h, compared with the non-treated control (Fig 2A and 2B). These results were similar to the effects on the migration of H23 and H292 cells, whereby H23 and H292 cells migration was also significantly decreased by 5 and 10 μM norcycloartocarpin at 24 and 48 h. We next determined the effect of norcycloartocarpin on the invasion and migration properties of lung cancer cells. Cell invasion was determined using a transwell Boyden chamber assay, whereby the cells were treated with various concentrations of norcycloartocarpin (1, 5, and 10 μM) for 24 h. We found that norcycloartocarpin was able to decrease the number of cells migrating and invading through the matrigel layer of the assay at 24 h in a dose-dependent manner, compared with the non-treated control (Fig 2C–2F). An invasion assay was also performed, and it was found that norcycloartocarpin could decrease the number of invaded A549, H460, H23, and H292 cells.

Anchorage-independent growth is an ability of cancer cells to grow independently on semisolid medium as soft agar and is a hallmark of carcinogenesis [64]. Soft-agar colony formation assay is widely used to estimate the anchorage-independent growth ability of cancer cells [65]. Here, human lung cancer cells were grown in soft agar with or without norcycloartocarpin for 14 days. The size and number of the growing cancer cell colonies were determined and calculated relative to those of the untreated control. The number of colonies on A549 cells were significantly reduced by norcycloartocarpin treatment at 5, and 10 μM by 82.6%, and 63.3%, respectively, and the percentage colony sizes in response to norcycloartocarpin at concentrations of 1, 5, and 10 μM were 69.6%, 52.0%, and 41.6%, respectively (Fig 2D and 2E). In addition, the results showed that the number of colonies on H460 (Fig 2D and 2E) cells were significantly reduced by norcycloartocarpin at 1, 5, and 10 μM by 94.0%, 87.3%, and 64.0%, respectively, and the percentage colony sizes in response to the compound at 1, 5, and 10 μM were 74.0%, 56.6%, and 42.0%, respectively. These results revealed that norcycloartocarpin at non-toxic concentrations could suppress the survival and growth of cancer cells in the detached condition. Consistent with these findings, the number of filopodia formation of A549 and H460 cells were significantly decreased by norcycloartocarpin treatment at 5, and 10 μM (Fig 2G and 2H).

### Norcycloartocarpin suppressed EMT via the suppression of the FAK/Akt signaling pathway

**Fig 3:**
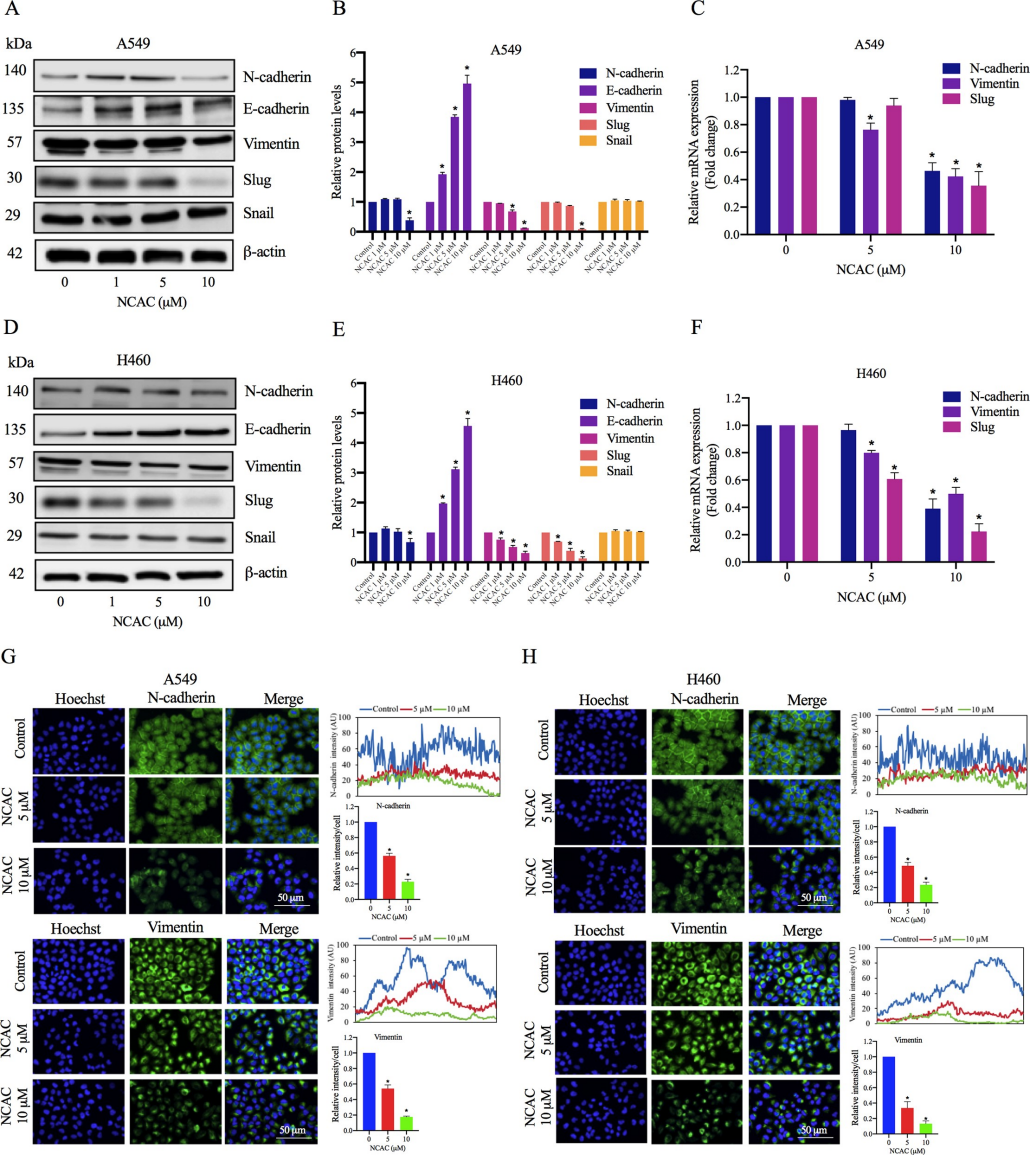
Effect of norcycloartocarpin (NCAC) on Epithelial to Mesenchymal Transition (EMT): A549 and H460 cells were treated with various concentrations of NCAC (0–10 μM) for 24 h. (**A**, **D**) The expression levels of EMT protein markers were investigated by Western blotting. (**B**, **E**) Blots were reprobed with β-actin to confirm equal loading of samples. The immunoblot signals were quantified by densitometry. (**C**, **F**) The mRNA expression levels of EMT markers (N-cadherin, vomentin and slug) were determined by quantitative real-time polymerase chain reaction. (**G**, **H**) A549 and H460 cells were treated with NCAC at indicated concentrations for 24 h. The cellular levels of N-cadherin, and vimentin were determined by immunofluorescence analysis. The fluorescence intensity was analyzed by ImageJ software. Data represent the mean ± SD (n = 3). *p<0.05 versus non-treated control.

EMT is a physiological process that has been recently associated with cancer progression and metastasis [66]. Therefore, the effect of norcycloartocarpin on EMT was first determined by the general cell morphology observation and Western blotting. A549 and H460 cells were treated with various non-toxic concentrations of norcycloartocarpin for 24 h. The EMT markers, namely N-cadherin, E-cadherin, vimentin, Slug, and Snail, were determined. We found that norcycloartocarpin significantly suppressed the levels of the EMT markers, such as N-cadherin, vimentin, and Slug, in A549 (Fig 3A and 3B) and H460 (Fig 3D and 3E) cells. Similar results were found that A549 and H460 cells, in which treatment with norcycloartocarpin resulted in a dramatic decrease the mRNA levels of N-cadherin, Vimentin, and Slug in a dose-dependent manner (Fig 3C–3F). We further confirmed the inhibitory effect of norcycloartocarpin on N-cadherin, and vimentin by immunofluorescence staining. We also found a significant reduction of the N-cadherin and vimentin proteins at concentrations of 5 and 10 μM in both A549 and H460 cells. Fig 3G shows that norcycloartocarpin significantly decreased the levels of N-cadherin and vimentin in A549 cells. Consistently, similar results from the immunofluorescence analysis in H460 cells were found, as shown in Fig 3H.

**Fig 4:**
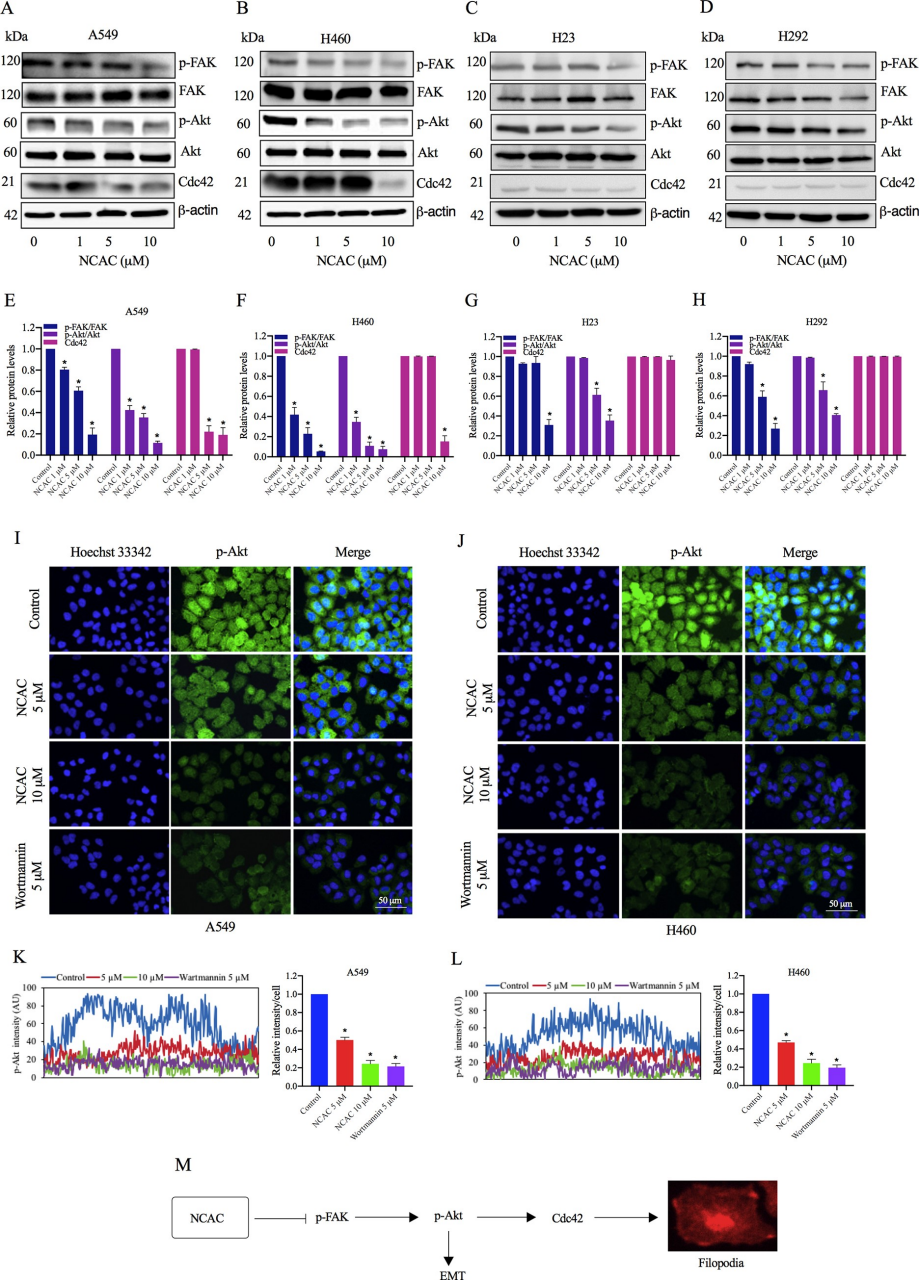
Effect of NCAC on Epithelial to Mesenchymal Transition (EMT) through inhibition of the FAK/Akt signaling pathway. A549, H460, H23, and H292 cells were treated with non-toxic concentrations of NCAC (1, 5, and 10 μM) for 24 h. (**A-H**) The expression levels of phosphorylated focal adhesion kinase (FAK) (Tyr397), phosphorylated Akt (Ser473), and Cdc42 were determined by Western blot analysis. (**I, K**) A549 and (**J, L**) H460 cells were treated with NCAC at indicated concentrations and wortmannin (5 μM) for 24 h. The cellular levels of p-Akt was determined by immunofluorescence analysis. The fluorescence intensity was analyzed by ImageJ software. (**M**) Schematic mechanism of NCAC in suppression of EMT and filopodia formation via inhibition of the FAK/Akt signaling pathway. Data represent the mean ± SD (n = 3). *p<0.05 versus non-treated control.

Moreover, the upstream regulatory cell signals of EMT and the controllers of cell migration, such as FAK, AKT, and Cdc42 were determined. Cdc42 has been shown to regulate cell behaviors in cancers that promotes filopodia formation and cell migration [67]. The results reveal that norcycloartocarpin was able to decrease the active form of FAK. The p-FAK (phosphorylated at Tyr397) was found to be significantly reduced in response to 1, 5, and 10 μM of norcycloartocarpin in A549 (Fig 4A–4E), H460 (Fig 4B–4F), H23 (Fig 4C–4G), and H292 cells (Fig 4D–4H). In addition, the activated Akt, p-Akt (phosphorylated at Ser473) were found to be suppressed in response to the compound treatment (Fig 4A–4H). Cdc42 level was also found to be significantly reduced in response to 5 and 10 μM of norcycloartocarpin in A549 cells; however, we found a significant reduction of the Cdc42 protein only at concentrations of 10 μM in H460 cells. The Akt inhibitory effect of norcycloartocarpin was confirmed by the immunofluorescence analysis in A549 and H460 cells (Fig 4I–4L). These results showed that norcycloartocarpin suppressed EMT and inhibited human lung cancer cell motility through the inhibition of the FAK/Akt signaling pathway.

### The activation of apoptosis cascade in norcycloartocarpin-treated lung cancer cells

**Fig 5:**
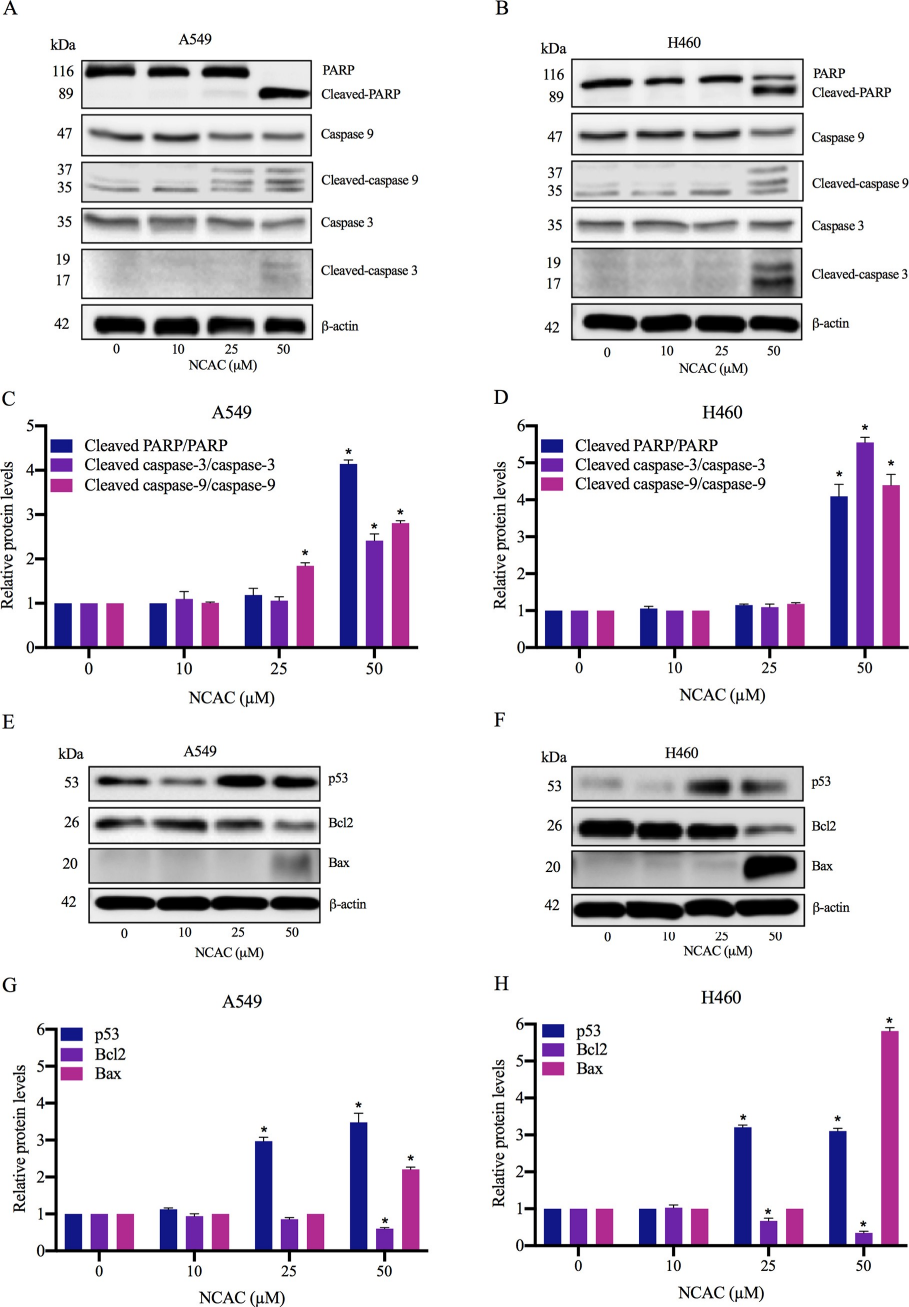
Apoptosis caspase cascade activated by norcycloartocarpin. The augmented level of cleaved caspase-9, an initiator caspase of apoptosis cascade, was demonstrated in lung cancer (**A**) A549 and (**B**) H460 cells cultured with 25–50 μM norcycloartocarpin (NCAC) for 24 h. (**C**) and (**D**) NCAC at 50 μM obviously up-regulated active form of caspase-3, cleaved caspase-3, in lung cancer A549 and H460 cells, respectively. Moreover, the enzymatic function of cleaved caspase-3 was also evidenced with the conversion of PARP to cleaved PARP in NCAC-treated cells. The alteration of Bcl-2 family proteins including reduced Bcl-2 and accumulated Bax associating with the up-regulation of p53 was notified in both (**E, G**) A549 and (**F, H**) H460 lung cancer cells incubated with NCAC at 50 μM for 24 h. The restoration of p53 and down-regulation of Bcl-2 was also presented in the cells after culture with 25 μM NCAC. Data are represented as mean ± SEM from three independent experiments. *p < 0.05 versus non-treated control cells.

The activation of apoptosis cascade in norcycloartocarpin-treated lung cancer cells was further verified via western blot analysis. Fig 5 respectively indicate the alteration of apoptosis maker proteins in human lung cancer A549 and H460 cells cultured with 10–50 μM norcycloartocarpin. The correspondent increase of activated caspase3 (Cleaved caspase-3) and degraded form of its substrate, cleaved PARP, was obviously noticed in the cells treated with 50 μM norcycloartocarpin for 24 h (Fig 5A–5D). Moreover, there was the higher expression of cleaved caspase-9, an initiator caspase in A549 and H460 cells after incubation with norcycloartocarpin at 25 and 50 μM as presented in Fig 5A and 5B, respectively. The up-regulated ratio of cleaved caspase-9/caspase-9 correlated well with apoptosis cell death found in norcycloartocarpin-treated lung cancer cells. Taken together, these results strongly support the apoptosis inducing effect of norcycloartocarpin in human lung cancer cells.

### Tumor suppressor p53 and relating proteins triggered by norcycloartocarpin

As caspase-9 is an initiator caspase relating to intrinsic apoptosis pathway [68], the modulating activity of norcycloartocarpin on Bcl-2 family proteins was further investigated. After culture with norcycloartocarpin at 25–50 μM, down-regulated Bcl-2 associating with the overexpression of tumor suppressor p53 protein was obviously demonstrated in both A549 (Fig 5E–5G) and H460 cells (Fig 5F–5H). Notably, these alteration of Bcl-2 and p53 level correlated to the activation of caspase-9 and apoptosis induction (Fig 5A–5D) that were also observed in these norcycloartocarpin-treated cells. Fig 5E and 5F indicate that Bax, an affected molecule of p53 was correspondently augmented in the cells treated with norcycloartocarpin at 50 μM. The modulation on up-stream regulatory protein mediating p53 function was also evaluated. Interestingly, the suppression on Akt survival signal indicated by the reduction of phosphorylated Akt (p-Akt)/Akt ratio was significantly observed in both A549 and H460 cells cultured with norcycloartocarpin.

### Molecular docking simulation revealed the norcycloartocarpin interactions with the Akt protein

**Fig 6:**
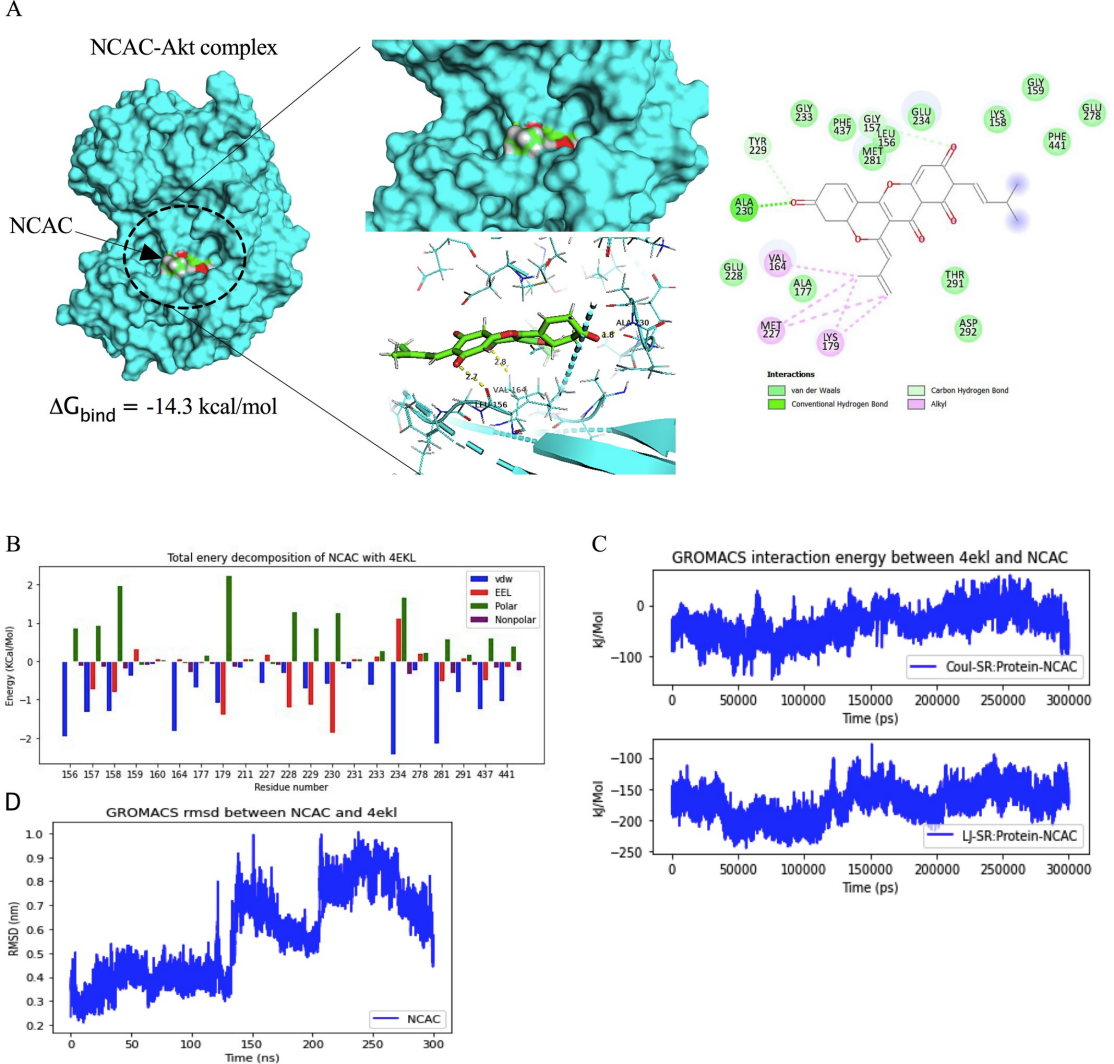
Docked model depicting interaction of norcycloartocarpin (NCAC) with Akt protein. (**A**) Binding mode and docking energy of NCAC bound to the binding site of Akt taken from the MD study. (**B**) Total energy decomposition of NCAC-Akt complex using the gmx_MMPBSA program. (**C**) The interaction energy between NCAC and Akt. (**D**) The RMSD plot for interaction of NCAC-Akt complex during 300 ns of molecular dynamic simulation.

The binding affinity of norcycloartocarpin to important Akt proteins and regulators crucial for EMT and apoptosis in cancer cells was investigated using molecular docking methods. The binding affinity score calculated from AutoDock vina was -14.3 kcal/mol. To estimate the binding affinity of norcycloartocarpin against the focused Akt protein, the molecular mechanics combined with the generalized Born surface area (MM/GBSA) method was applied on the 200 molecular dynamics (MD) snapshots extracted from the last 200-ns simulations. The total contributing amino acids of norcycloartocarpin-Akt complex is shown in Fig 6A and S1 Video, the key binding residues ALA230, VAL164, LEU156, and MET281 involved in norcycloartocarpin binding were identified to those of the Akt inhibitors. Interestingly, the ligand binding mode of Akt model shared a structurally-related characteristic, in which the aromatic moiety of all studied compounds approached the key residue ALA230. The binding free energy (Δ*G*_bind_) together with its energy components are summarized in Table 1. By considering Akt model, the Δ*G*_bind_ of norcycloartocarpin-Akt complex was -12.52 kcal/mol. Due to the polar structure of norcycloartocarpin (Fig 6B), the molecular mechanics energy (Δ*E*_MM_) revealed that van der Waals interaction (Δ*E*_vdW_) was the main force driving protein-ligand complexation (Δ*E*_vdW_ of -46.48 ± 2.35 kcal/mol) for norcycloartocarpin-Akt complex. In contrast, the electrostatic attraction (Δ*E*_ele_) was found to mainly contribute toward norcycloartocarpin-Akt (Δ*E*_ele_ of -13.20 ± 7.34 kcal/mol) complex. Ligand positional RMSD was generated to evaluate the binding stability of norcycloartocarpin-Akt complex, indicating lower or high stability (Fig 6D). The residue decomposition free energy calculation based on the MM/GBSA method was used to investigate the crucial amino acid residues involved in ligand binding within the ATP-binding pocket of Akt.

**Table 1:** The MM/GBSA (Δ*G*_bind_) and its energy components (kcal/mol) of the norcycloartocarpin-Akt complex.

	Norcycloartocarpin-Akt (Δ*G*_bind_)
Δ*E*_ele_	-13.20 ± 7.34
Δ*E*_vdW_	-46.48 ± 2.35
Δ*E*_MM_	-59.69 ± 7.92
Δ*G*_solv,non-polar_	-6.02 ± 0.12
Δ*G*_solv,polar_	34.02 ± 6.57
Δ*G*_total_	-31.69 ± 2.77
-TDS	19.17
Δ*G*_bind_	-12.52

## Discussion

In searching for the effective chemotherapeutic agents, current researches have indicated the promising anticancer activity of various compounds from natural resources. Not only high anticancer potency but also human safety profile advocates the development of natural extracts as a novel chemotherapy [40]. Recently, anti-migratory activity of compounds extracted from *A*. *gomezianus*, a Thai medicinal plant, has been reported in human lung cancer cells [42, 43]. Additionally, the selective cytotoxicity of norcycloartocarpin, a flavonoid derived from *A*. *gomezianus* which was evidenced with the higher IC_50_ (approximately 2-fold) in human dermal papilla cells compared with various human lung cancer cells was firstly revealed in this study (Fig 1C). Chemotherapy-induced alopecia critically lessen quality of life and can lead to low drug compliance in lung cancer patients [69, 70]. Therefore, the obtained information suggests that *A*. *gomezianus* extracts especially norcycloartocarpin is a potential anticancer agent with low toxicity to normal cells for treatment of lung cancer.

Metastasis is a key process of cancer cells migrating from the primary site to secondary locations and is a leading cause of death worldwide [71]. Epithelial-to-mesenchymal transition (EMT) is a critical factor for the successful spread of human cancer cells that results in the loss of epithelial characteristics and acquiring mesenchymal properties including invasiveness, motility, and resistance to apoptosis, as well as leading to successful metastatic colonization [72, 73]. Herein, we found that norcycloartocarpin suppresses cell migration and EMT in lung cancer cells (Figs 2 and 3). In direct immunofluorescence studies, we also found a significant reduction of the N-cadherin and vimentin proteins at concentrations of 5–10 μM in norcycloartocarpin-treated lung cancer cells. Moreover, p-FAK (phosphorylated at Tyr397), p-Akt (phosphorylated at Ser473), and Cdc42 were found to be significantly reduced in response to non-toxic dose of norcycloartocarpin in A549, H460, H23, and H292 cells (Fig 4). Previous reports indicated that phosphorylated FAK and Akt have been reported to serve a significant role in the regulation of cell proliferation, survival, adhesion, and migration [74–76]. Thus, we suggested that the reduction of phosphorylated FAK and Akt may mediate the inhibitory effect of norcycloartocarpin on cell migration and EMT of lung cancer cells via the inhibition of the FAK/Akt signaling pathway.

Accordingly, Akt is a regulator that affect many cellular functions including cell growth and survival, differentiation, migration, metabolism, and metastasis [77, 78]. Thus, the circled part of norcycloartocarpin was deployed as a docking site for examination of the binding properties. After investigation, the results revealed that the binding affinity of norcycloartocarpin was high, which indicated their ability to bind properly with Akt protein in the ATP-binding site (Fig 6A). The predicted binding models indicate that norcycloartocarpin has multiple interactions with surrounding residues, including ALA230, VAL164, LEU156, and MET281. Similarly, Chuang *et al*. reported that compounds a46 and a48 are hydrogen-bonded to residues ALA230, THR211 and ASP292. These compounds have multiple hydrophobic interactions with surrounding residues, such as LEU156, VAL164, MET227, TYR229, PHE237, MET281, PHE438 and PHE442 [79]. Currently, the development of novel small-molecule Akt inhibitors is mainly focused on the active compounds that can bind to the ATP-binding site of Akt protein [79, 80]. Several classes of ATP-competitive Akt inhibitors have been reported, such as AZD5363 [81], GSK690693 [82], and LY2780301 [83]. Especially, norcycloartocarpin may serve as useful lead agent for further development of anticancer compounds.

The recurrence of tumor pathology has been gradually documented in lung cancer patients after administration of conventional chemotherapy [84–86]. The capability to reproduce a tumor colony of lung cancer cells after culture with norcycloartocarpin was assessed in this study via colony forming assay. The inhibitory effect on colony formation was correspondent with the suppression of p-Akt/Akt survival signal in A549, H460, H23, and H292 cells derived after norcycloartocarpin treatment (Fig 4). According to the modulation on both survival and apoptosis machinery, PI3K/Akt pro-survival signaling is one of the most valuable targets for lung cancer treatment [87]. The relevance of pro-survival signal has been evidenced with the overexpression of Akt in lung cancer cells reported chemotherapeutic failure [88]. Moreover, the diminution of Akt survival signal could enhance drug sensitivity, prevent tumor recurrence and improve poor prognosis in various cancers [89–91]. The inhibition of Akt associating with p53 activation and down-regulation of anti-apoptosis have been well recognized [36–38, 92]. It has been reported that p-Akt restrains the expression of p53 in human lung cancer cells [93]. Correspondently, the augmentation of p53 level in human lung cancer cells presented in this study might be a consequence of the suppressive activity of norcycloartocarpin on p-Akt/Akt signalling (Fig 4).

Aggressive features including chemoresistance of human lung cancer cells crucially contribute to the therapeutic failure of current anticancer drugs [11, 94]. Lung cancer cells isolated from clinical specimens overexpressed anti-apoptosis Bcl-2 protein and possessed low susceptibility to available chemotherapy [95]. The down-regulated Bcl-2 (Fig 5) associating with apoptosis induction which was indicated by chromatin condensation and/or nuclei fragmentation and activation of caspase-9 (Fig 5) were presented in lung cancer cells after culture with 25 μM norcycloartocarpin for 24 h. Attractively, the inhibition of Bcl-2 has been proposed as an effective strategy for novel anticancer treatment [96–98]. The augmented level of p53, an up-stream mediator of apoptosis was correlatedly presented in norcycloartocarpin-treated cells. Activation of p53 also results in the reservation of Bax, a pro-apoptosis protein that play an important role on apoptosis initiation in lung cancer cells at advance stage [99]. Indeed, the irregular expression of p53, Bcl-2 and Bax have been considered as poor prognosis markers in lung cancer patients [95, 100]. The promising anticancer activity of norcycloartocarpin was clearly demonstrated with the restoration tumor suppressive function of p53 consequence with accumulation of Bax, reduction of Bcl-2 and eventually apoptosis induction in human lung cancer cells. Although apoptosis inducing effect was certainly notified in lung cancer cells treated with 25–50 μM norcycloartocarpin, Akt survival signal and new colony formation were dramatically repressed at the lower concentration (10 μM). As a pivotal modulator regulating diverse cancer behaviors including anti-apoptosis, survival, proliferation, and metastasis, the inhibition on Akt signaling pathway at a non-toxic concentration in normal cells would emphasize the therapeutic benefits of norcycloartocarpin for treatment of lung cancer.

## Conclusion

**Fig 7:**
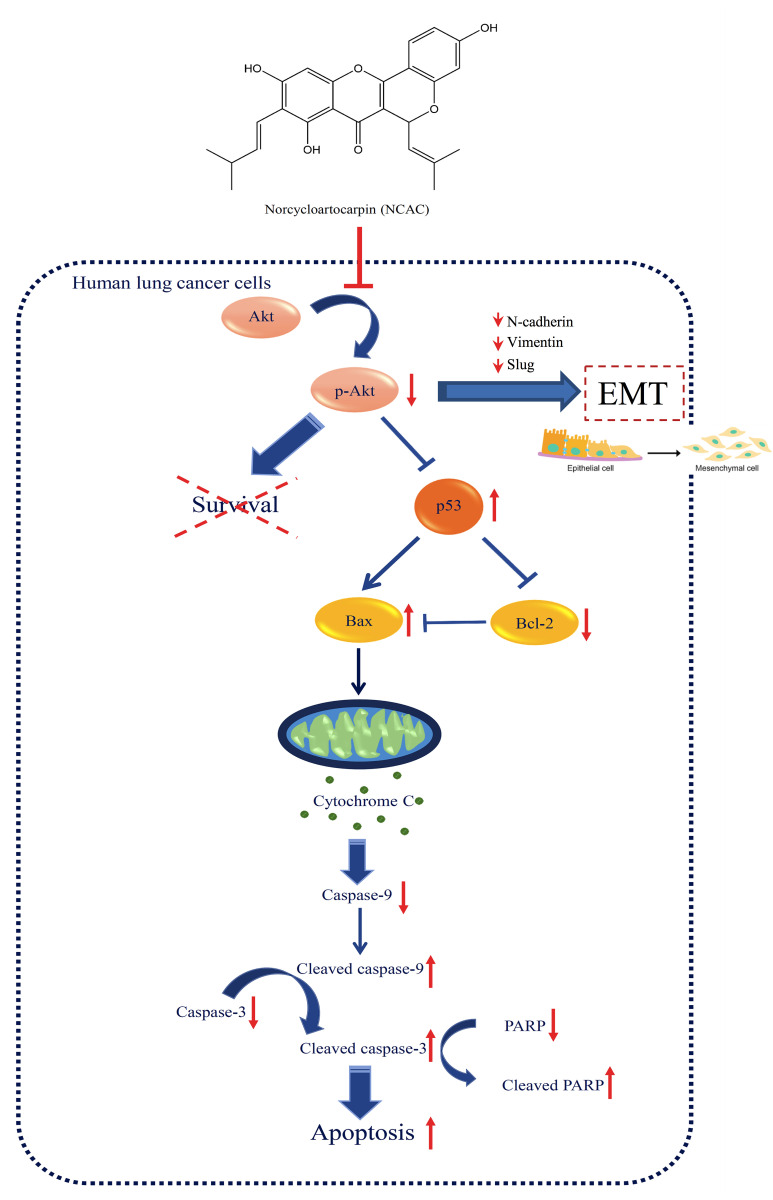
The proposed regulatory pathway involving in anticancer activity of norcycloartocarpin in human lung cancer cells. The mechanism of norcycloartocarpin in suppression of the migration and EMT signals in lung cancer cells. Norcycloartocarpin triggers apoptosis via Akt-modulating p53 activation following with down-regulation of Bcl-2 and increase of Bax. Moreover, the suppression on p-Akt/Akt survival signal mediated by norcycloartocarpin resulted in the restraint of survival ability and colony formation in human lung cancer cells.

In conclusion, norcycloartocarpin extracted from *A*. *gomezianus* could effectively suppress cell migration and EMT, which consequently decrease EMT markers and downstream migratory proteins, as concluded in the summarized schematic figure (Fig 7). We also found that norcycloartocarpin activate apoptosis in human lung cancer cells through suppression of Akt-mediated survival consequence with the modulation on p53 and apoptosis regulating proteins, Bcl-2 and Bax. Accumulating with the selective cytotoxicity against various human lung cancer cells, norcycloartocarpin could be considered as a promising anticancer agent for further development of a novel chemotherapy.

## Supporting information

S1 Video Docked model depicting interaction of NCAC-Akt complex. (MOV)

S1 File (PDF)
