# Supplementary figures and images for: Norcycloartocarpin targets Akt and suppresses Akt-dependent survival and epithelial-mesenchymal transition in lung cancer cells
Source: PLoS One. 2021 Aug 12;16(8):e0254929. doi: 10.1371/journal.pone.0254929 (PMC8360371; doi:10.1371/journal.pone.0254929)

Figure 3A

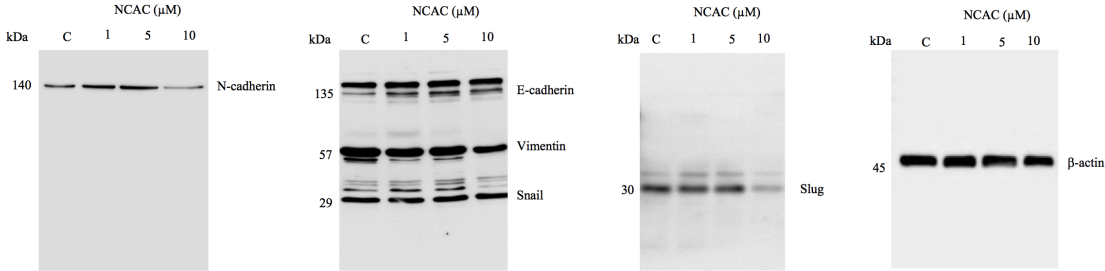

Figure 3D

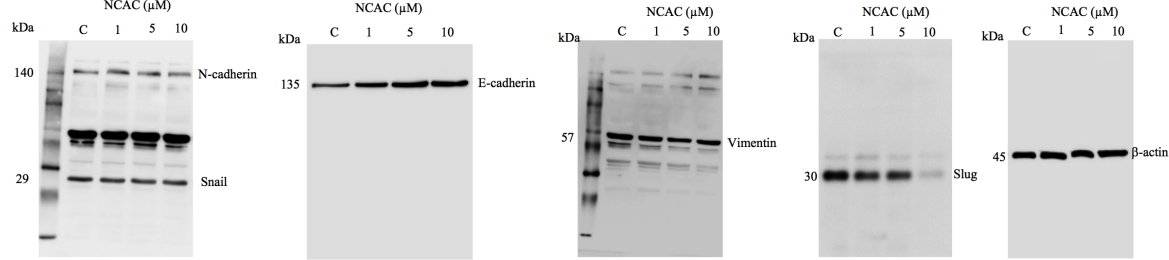

Figure 4A

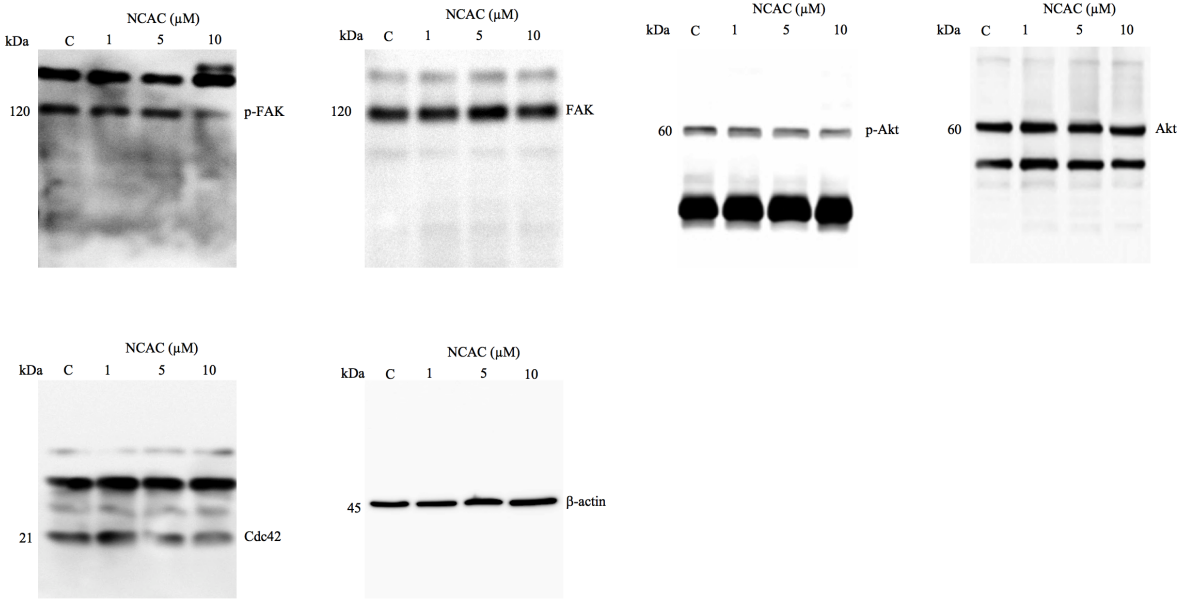

Figure 4B

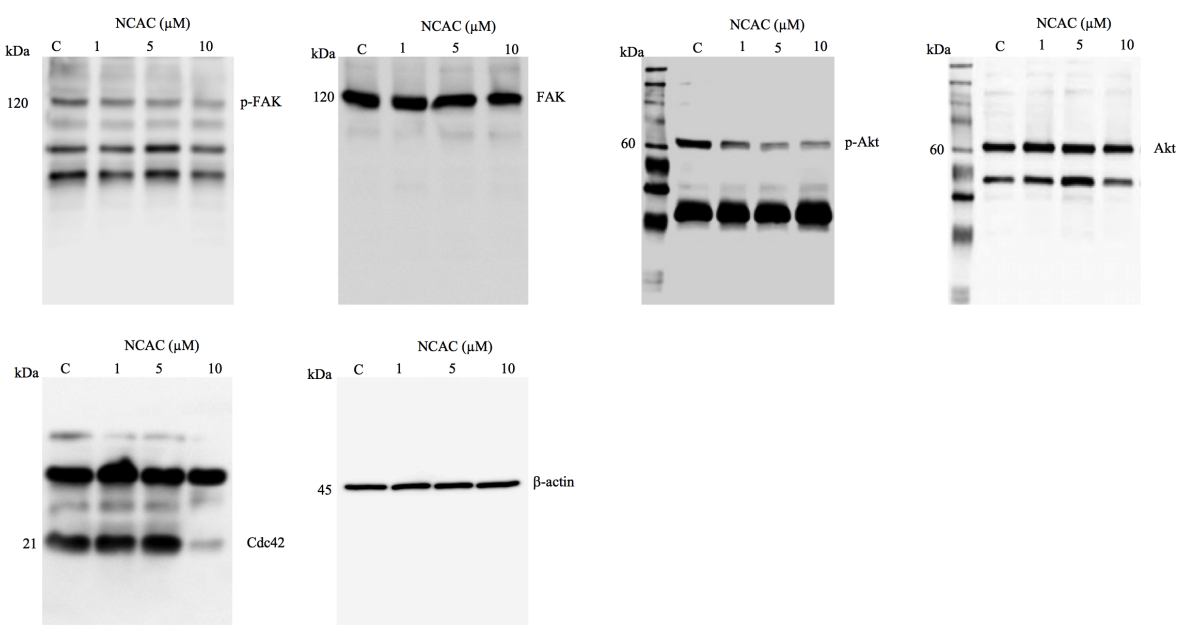

Figures 4C and D

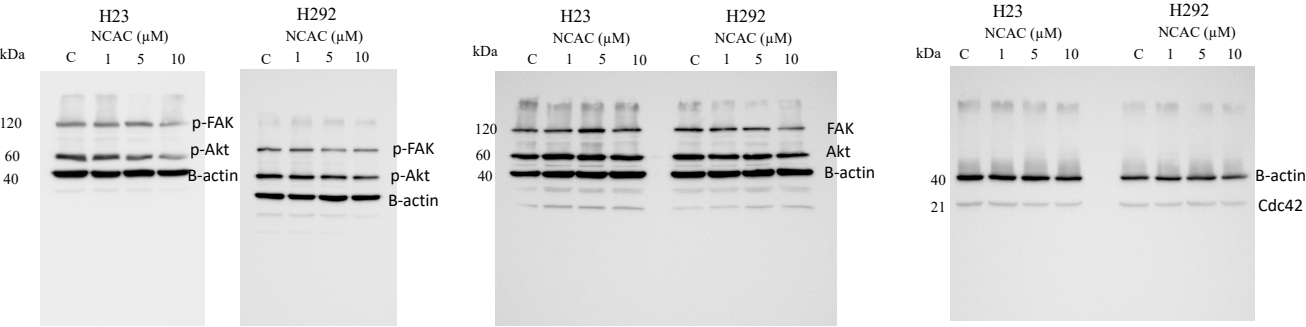

Figure 5A

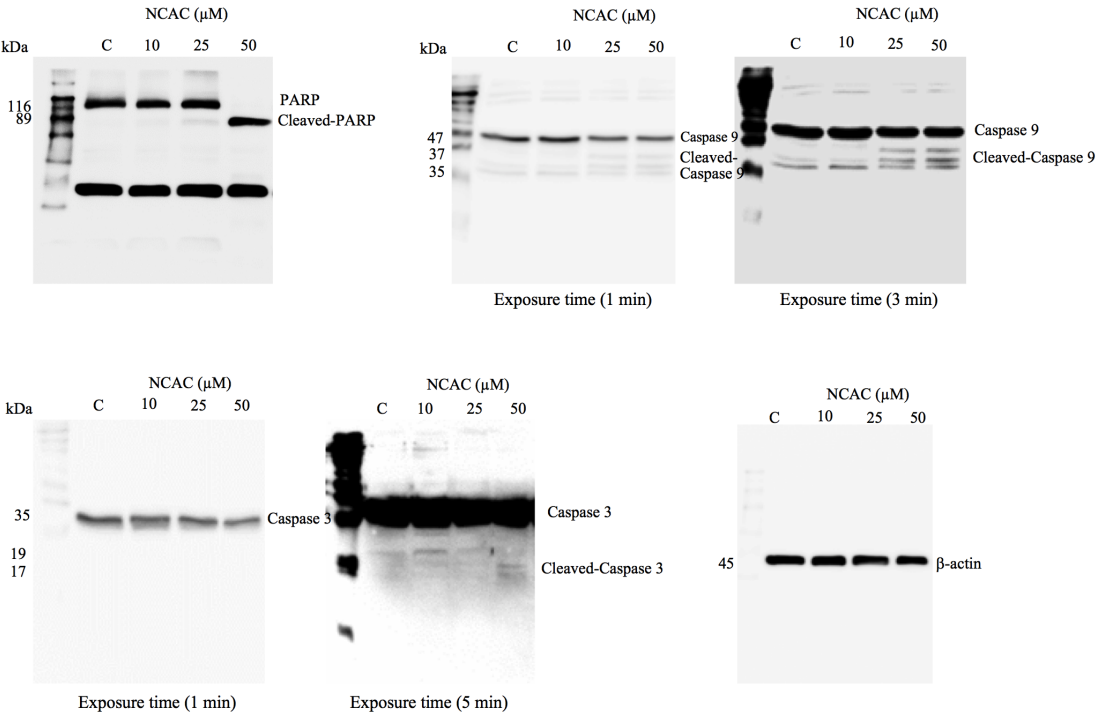

Figure 5B

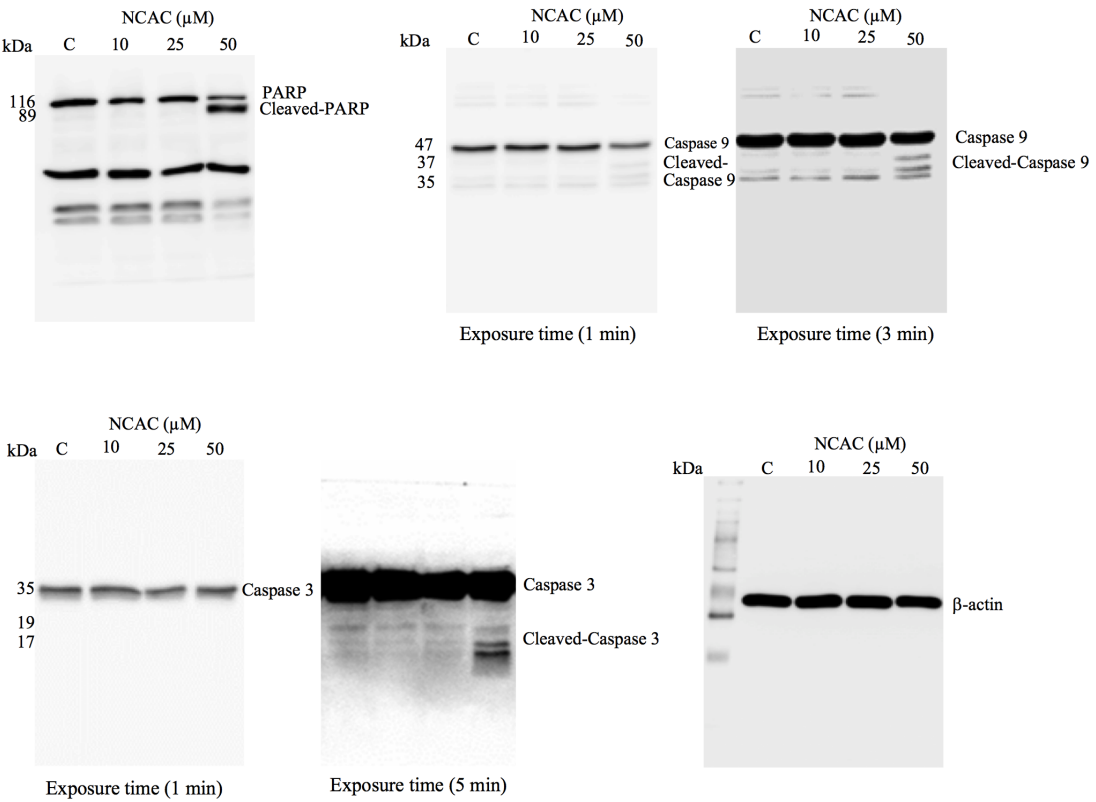

Figure 5E and F

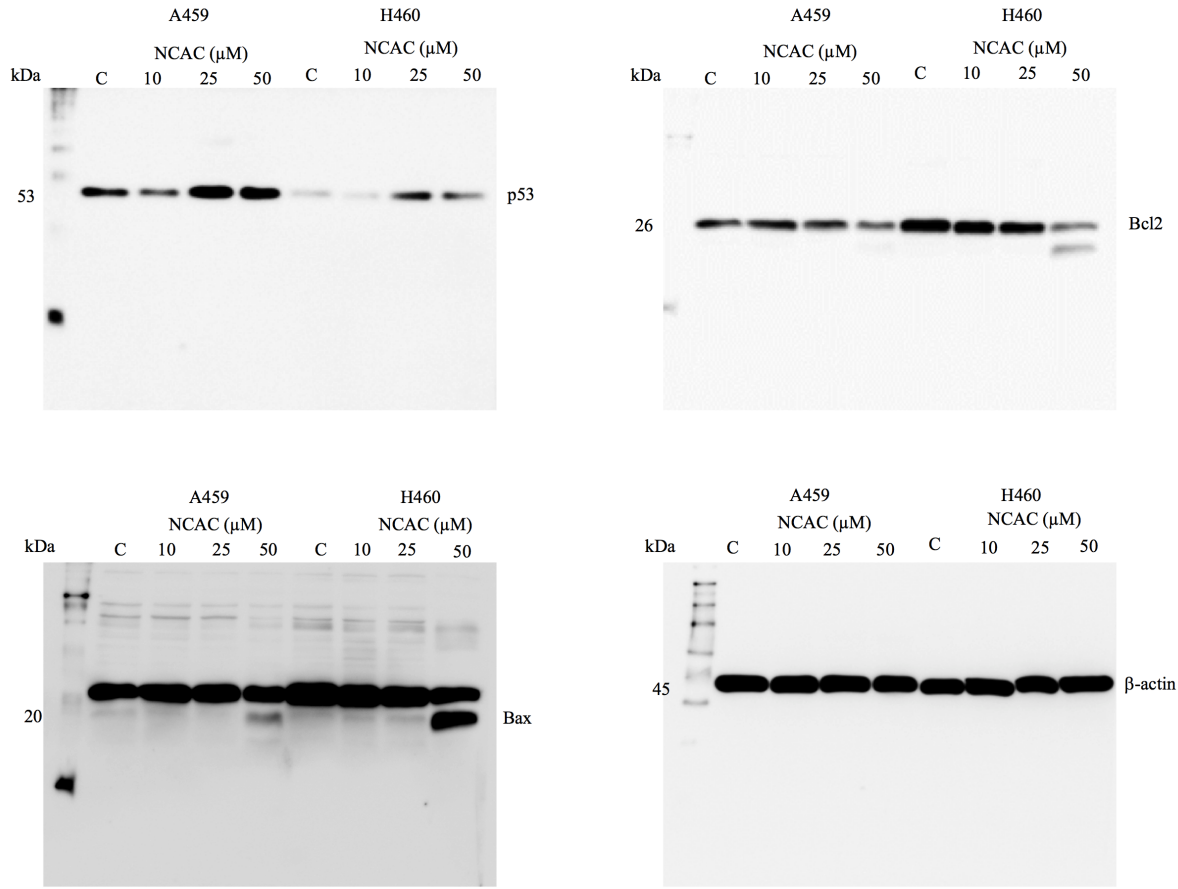

Supplement: S1 File — (PDF) [file pone.0254929.s002.pdf]
